# The association between muscle strength and executive function in children and adolescents: Based on survey evidence in rural areas of China

**DOI:** 10.3389/fpsyg.2022.1090143

**Published:** 2023-01-06

**Authors:** Qiang Zeng, Xin Hu, Yujie Wang

**Affiliations:** ^1^School of Physical Education, Huanghuai University, Zhumadian, China; ^2^Department of Public Education, Zhumadian Vocational and Technical College, Zhumadian, China

**Keywords:** children and adolescents, muscle strength, executive function, China, rural areas

## Abstract

**Background:**

In recent years, muscle strength in children and adolescents has continued to decline, especially in rural areas. Executive function as a higher function of the brain, is closely related to the future achievement of children and adolescents. For this reason, this study analyzed the correlation between muscle strength and executive function in children and adolescents in rural areas of China to better promote the development of muscle strength and executive function.

**Methods:**

Demographic factors, muscle strength, and executive function were tested in 1,335 children and adolescents in rural China using a three-stage stratified whole-group sampling method. One-way ANOVA and chi-square test were used to compare the differences in executive function among children and adolescents with different muscle strengths. Multiple linear regression analysis and logistic regression analysis were used to analyze the association that exists between muscle strength and executive function.

**Results:**

When comparing the inconsistently, congruent, 1back, 2back, conversion, size parity, and cognitive flexibility response times of children and adolescents with different muscle strength indexes in rural China, the differences were statistically significant (*F* = 46.592, 45.610, 10.809, 32.068, 24.095, 19.260, 11.501, *p* < 0.001). Logistic regression analysis was performed using children and adolescents with muscle strength index >P80 as the control group. The results showed that the risk of 1back dysfunction was higher (OR = 1.764, 95% CI:1.094, 2.843) in children and adolescents with muscle strength index <P20 (*p* < 0.05). The risk of 2back dysfunction was higher in children and adolescents with a muscle strength index <P20 (OR = 2.129, 95% CI:1.329, 3.410) (*p* < 0.01). Compared with children and adolescents with muscle strength index >P80. The muscle strength index <P20 group had a higher risk of cognitive flexibility dysfunction (OR = 1.820, 95% CI:1.111, 2.982) (*p* < 0.05).

**Conclusion:**

There is a association between muscle strength and executive function in children and adolescents in rural areas of China. Those with higher muscle strength have shorter executive function reaction times and are at lower risk of developing executive dysfunction. Future measures should be taken to improve muscle strength and executive function levels in children and adolescents in rural areas to promote healthy physical and mental development.

## 1. Introduction

Muscle strength is an important component of physical fitness (Kell et al., 2001; Stodden et al., 2017). Inadequate muscle strength in children and adolescents is recognized as an important marker of poor health (Ortega et al., 2008; Peterson and Krishnan, 2015). Evidence suggests that muscle strength is a strong marker of current and future cardiovascular risk and is negatively associated with all-cause mortality, independent of cardiorespiratory fitness levels (Ortega et al., 2012; Fraser et al., 2016; Ramirez-Velez et al., 2016). Moreover, inadequate muscle strength in children and adolescents is a new risk factor for suicide (Ortega et al., 2012). A meta-analysis showed that the combined risk ratio (HR) for mortality in the weakest and strongest grip groups was 1.67 (Cooper et al., 2010). The number of 1-min sit-ups was negatively associated with mortality (Katzmarzyk and Craig, 2002). Of concern is the long-term trend of decreasing muscle strength levels in young people that has been reported across the globe (Sandercock and Cohen, 2019; Duric et al., 2021; Fuhner et al., 2021). There are also studies that confirm that less than two-fifths of Chinese children and adolescents met the World Health Organization muscle-strengthening exercise recommendations (Xin et al., 2022). Therefore, considering the physical and mental health benefits of muscle strength, further attention needs to be paid to the muscle strength profile of Chinese children and adolescents.

Executive function refers to the top-down control of behavior through the use of its core components, such as inhibit function, refresh memory function (1back, 2back), and cognitive flexibility (Miyake and Friedman, 2012). Normal performance of executive function is associated with physical health, mental health, academic performance and productivity, and career achievement (Diamond, 2013). The development of executive function in children and adolescents is influenced by multiple factors, including the educational environment, dietary behavior, different environmental exposures, and physical activity levels (Zysset et al., 2018; Zeng et al., 2022). Evidence from neurotypical cohorts suggests that high performance on executive function tasks in children and adolescents is associated with high cardiorespiratory fitness and muscle strength (Ludyga et al., 2021). Recent studies have found a strong association between executive function and muscle strength (Liu-Ambrose et al., 2010; Arts et al., 2016), and resistance training has been found to have positive and significant effects on improving functional brain plasticity, executive function, and response inhibition (Yoon et al., 2017). It has also been confirmed that physical exercise enhances cognition and brain function, plays a positive role in preventing neurodegenerative diseases and neurological decline, and is helpful for cognitive brain function (Kramer and Erickson, 2007). This shows that foreign studies on the association between muscle strength and executive function are increasing year by year, but the study population is mostly middle-aged and elderly people or special children and adolescents with diseases, and there are fewer studies on the correlation between muscle strength and executive function in normal children and adolescents (Ludyga et al., 2021).

A further review of previous studies revealed some shortcomings in this area of research. First, fewer studies have been conducted using comprehensive muscle indices. Muscle strength tests are an important part of physical fitness tests in many countries and regions in the world today. The most commonly used simple muscle strength test indices in Chinese children and adolescents’ physical fitness tests are grip strength, pull-ups, standing long jump, and sit-ups. Grip strength and pull-ups are measures of upper limb muscle strength, standing long jump is a common indicator of lower limb body muscle strength, and sit-ups are valid indicators in abdominal strength tests (Yin et al., 2018). However, in many studies, indicators of individual general muscle strength are evaluated with grip strength (Wind et al., 2010), without testing two or more body parts, and a single test cannot fully represent whole-body muscle strength. Therefore, there is a need to investigate the association of whole-body muscle strength aspects on executive function. Second, China is a developing country with highly uneven regional development. There are large disparities between urban and rural children and adolescents in terms of socioeconomic status, educational resources, nutritional status, and physical activity, and fewer previous studies have been conducted on children and adolescents in rural areas. Third, previous studies have found urban-rural differences in muscle strength test results (Liu et al., 2019). In addition, previous analyses of the association between muscle strength and executive function have included limited control for covariates. However, the literature suggests that factors such as parental education, dairy products (Knight et al., 2015), sleep quality (Sewell et al., 2021), and BMI all have an effect on brain executive function, so adjustments need to be given in the analysis to obtain more reliable research evidence (Rollins et al., 2021).

In view of the current status of previous studies, our study tested upper limb, abdominal, and lower limb muscle strength in children and adolescents from rural areas of China and calculated muscle strength indices according to age and gender to provide a comprehensive picture of the muscle strength levels of children and adolescents. Executive function was also tested to analyze the association that exists between muscle strength and executive function. To our knowledge, this is the first study in China to explore the association between muscle strength and executive function in rural children and adolescents. Our study provides a reference and help for the improvement of muscle strength and executive function in Chinese children and adolescents.

## 2. Materials and methods

### 2.1. Participants and procedures

**FIGURE 1:**
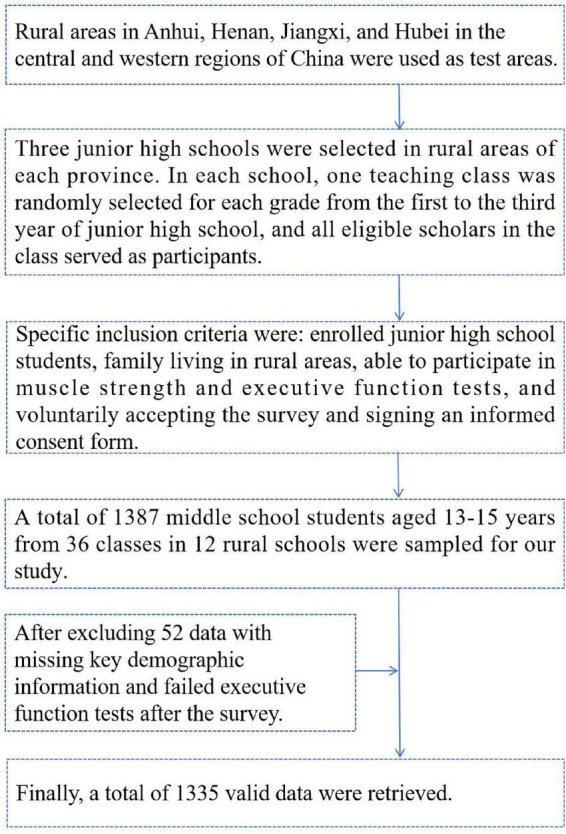
Flow chart of the sampling of participants in this study.

We used a three-stage stratified whole-group sampling method to draw subjects. In the first stage, rural areas in Anhui, Henan, Jiangxi, and Hubei in the central and western regions of China were used as the provinces for testing. In the second stage, three junior high schools in rural areas were selected in each province. In the third stage, in each school, one teaching class in each grade from the first to the third grade was randomly selected in whole groups, and the students in the class who met the requirements were used as test subjects for this study. Specific inclusion criteria were: junior high school students enrolled in school, family domicile and residence in a rural area, physical ability to participate in tests of muscle strength and executive function, and voluntary acceptance of the survey and signing of an informed consent form. Ultimately, a total of 1,387 middle school students aged 13–15 years from 36 classes in 12 rural schools were sampled for our study, and after excluding 52 data with missing key demographic information and failed executive function tests after the survey, a total of 1,335 valid data were retrieved. The average age of the participants was (14.15 ± 0.84) years. The specific sampling procedure is described in Figure 1.

Written informed consent was obtained from the participants themselves, their parents or guardians for the investigation of our study. The study was approved by the Human Ethics Committee of Huanghuai University (202105012). The study complied with the ethical procedures and requirements.

### 2.2. Muscle strength

Our study muscle strength tests include grip, pull up, sit ups, standing long jump a total of four indicators. Both boys and girls tested grip, standing long jump boys tested pull up and girls tested sit ups. The test results were standardized according to gender and age group. The standardized results of each subject were summed to obtain the muscle strength index (MSI), which reflects the overall muscle strength level of the subjects. Grip, pull up, sit ups and standing long jump were tested according to the testing instruments and methods required by the China National Student Physical Fitness Survey. Grip was accurate to 0.1 kg, pull up and sit ups were accurate to 1 time, and standing long jump was accurate to 0.1 cm. The grip strength test requires the arm to drop naturally and the strong side of the arm is used for the test. The standing long jump is performed by using a standing jump.

### 2.3. Executive function

We studied tests of executive functions including inhibit function, refresh memory function (1back, 2back), and cognitive flexibility the test methods for each specific executive function are described in a previous study (Yang et al., 2021). The test paradigm for executive functions has been validated in several studies and is suitable for testing Chinese children and adolescents (Wang et al., 2020; Zhang et al., 2022). The testing of the executive functions was done on a computer. The tests were performed using E-Prime software version 1.1. The executive functions included inhibit function, refresh memory function (1back, 2back), and cognitive flexibility inhibit function was evaluated using the Eriksen flanker task, and consistent and inconsistent response times (RT, ms) were recorded as test results. The difference between the two times was the result of the inhibit function. 1-back and 2-back tasks were used to assess the refresh memory function the shorter the reaction time, the better the performance of the refresh function. The more-odd shifting function was used to evaluate the cognitive flexibility of the subjects the shorter the reaction time, the better the cognitive flexibility. Participants were tested on a computer in a bright and spacious classroom environment with quiet surroundings, requiring them to concentrate on the test. Participants were required to avoid staying up late, drinking alcohol, and consuming excitatory drinks the day before the test.

### 2.4. Covariate

The covariates in our study included age, sex, only child, father’s education level, mother’s education level, breakfast, dairy products, duration of sleep, BMI, moderate and vigorous physical activity (MVPA). The father’s education level and mother’s education level were divided into two groups: junior high school and below, high school and above. Breakfast and dairy products were divided into two groups: ≤3times/week, ≥ 4times/week. Duration of sleep was divided into two groups: ≥8 h/day, <8 h/day. The body mass index (BMI) was calculated based on the tested height and weight, and the formula was weight (kg)/height (m)^2^. MVPA was investigated by the subjects based on their average daily MVPA in the past 7 days. MVPA time included fast running, heavy lifting, ball games, ice skating, skiing, etc. when they felt out of breath. The average daily MVPA time in min/d was calculated based on the number and average time of MVPA performed per day over the past 7 days.

### 2.5. Quality control

Our research survey consisted of trained and tested teachers and graduate students divided into four groups that entered different provincial schools at the same time to conduct the survey. A uniform instructional language was used to explain the requirements of the survey and test to the participants before the survey. Questionnaires were distributed on the spot and filled out on the spot, and questions were answered promptly in the process of filling them out. The questionnaires were collected on the spot after completion. The tests of executive functions were explained and demonstrated by the instructor and then tested by the participants on the computer. The tests were filled out using anonymous numbers.

### 2.6. Statistical analysis

Comparison of different muscle strength index classifications of children and adolescents in rural areas of China was performed using one-way methodological analysis (continuous variables) and chi-square test (count data). Count data were expressed as percentages and continuous variables were expressed as means and standard deviations (M ± SD). The comparison of executive function response times of children and adolescents with different muscle strength index was performed by one-way ANOVA. The association analysis between muscle strength index and executive function was studied by multiple linear regression analysis. Muscle strength index <P20 was used as the reference group for the analysis. Model 1 was the unadjusted variable, model 2 adjusted for age, sex, only child, father’s education level, and mother’s education level. Model 3 was adjusted for breakfast, dairy products, duration of sleep, BMI, and MVPA on the basis of Model 2. In order to further analyze the association between muscle strength index and executive function, we also conducted logistic regression analysis. Based on the standard deviation of the mean values of each subfunction of executive function by age and gender, children and adolescents with a test response higher than 1 standard deviation were defined as having executive dysfunction, and this was used to classify the presence or absence of executive dysfunction. Logistic regression analysis was performed with the muscle strength index >P80 group as the reference group, adjusted for different variables. The data were analyzed using SPSS25.0 (IBM Inc., Armonk, NY, USA) software, with α = 0.05 as the level of the two-sided test.

## 3. Results

We investigated demographic variables, grip strength, sit-ups, pull-ups, standing long jump and executive function in 1,335 (boys 672, 50.3%) children and adolescents aged 13–15 years in rural areas of China with a mean age of (14.15 ± 0.84) years.

Regarding the comparison of muscle strength index among different categories of children and adolescents in rural China, the differences were statistically significant when comparing the detection rates of different muscle strength index classifications in only child and sleep (χ^2^ = 7.638, 54.800, *p* < 0.05). In terms of BMI and MVPA, the differences were statistically significant when comparing different muscle strength index classifications (*F* = 69.418, 6.717, *p* < 0.01) (Table 1).

**TABLE 1:** Comparison of muscle strength index among different categories of children and adolescents in rural areas of China.

Sort	Muscle strength index classification			Total sample	χ^2^-value/*F*-value	*P*-value
	**<P20**	**P20–80**	**>P80**			
N	266 (19.9)	802 (60.1)	267 (20.0)	1,335		
Age (years)	14.09 ± 0.84	14.16 ± 0.84	14.19 ± 0.84	14.15 ± 0.84	1.025	0.359
**Sex**						
Boys	134 (50.4)	400 (49.9)	138 (51.7)	672 (50.3)	0.263	0.877
Girls	132 (49.6)	402 (50.1)	129 (48.3)	663 (49.7)		
**Only child**						
No	192 (72.2)	584 (72.8)	216 (80.9)	992 (74.3)	7.638	0.022
Yes	74 (27.8)	218 (27.2)	51 (19.1)	343 (25.7)		
**Father’s education level**						
Junior high school and below	134 (50.4)	388 (48.4)	128 (47.9)	650 (48.7)	0.394	0.821
High school and above	132 (49.6)	414 (51.6)	139 (52.1)	685 (51.3)		
**Mother’s education level**						
Junior high school and below	153 (57.5)	493 (61.5)	148 (55.4)	794 (59.5)	3.560	0.169
High school and above	113 (42.5)	309 (38.5)	119 (44.6)	541 (40.5)		
**Breakfast**						
≤ 3times/week	77 (28.9)	191 (23.8)	55 (20.6)	323 (24.2)	5.221	0.074
≥ 4times/week	189 (71.1)	611 (76.2)	212 (79.4)	1012 (75.8)		
**Dairy products**						
≤ 3times/week	57 (21.4)	200 (24.9)	77 (28.8)	334 (25.0)	3.908	0.142
≥ 4times/week	209 (78.6)	602 (75.1)	190 (71.2)	1001 (75.0)		
**Sleep**						
≥ 8 h/day	73 (27.4)	403 (50.2)	152 (56.9)	628 (47.0)	54.800	<0.001
< 8 h/day	193 (72.6)	399 (49.8)	115 (43.1)	707 (53.0)		
BMI (kg/m^2^)	19.97 ± 2.66	19.83 ± 2.53	20.42 ± 2.05	19.98 ± 2.48	69.418	0.003
MVPA (min/day)	35.61 ± 18.55	40.41 ± 23.57	42.38 ± 22.38	39.85 ± 22.52	6.717	0.001
Grip	23.60 ± 6.07	29.38 ± 7.57	38.11 ± 9.68	29.98 ± 9.06	237.697	<0.001
Pull up	1.00 ± 1.34	2.57 ± 1.90	5.78 ± 4.21	2.91 ± 2.93	135.994	<0.001
Sit ups	21.42 ± 6.37	30.13 ± 7.35	37.34 ± 6.22	29.80 ± 8.57	172.062	<0.001
Standing long jump	150.38 ± 22.75	173.71 ± 28.06	197.94 ± 30.86	173.91 ± 31.48	196.644	<0.001

N, number; BMI, body mass index; MVPA, moderate and vigorous physical activity.

Our study showed that the inconsistently, congruent, 1back, 2back, conversion, size parity, and cognitive flexibility response times of children and adolescents with different muscle strength indexes in rural China were compared, the differences were statistically significant (*F* = 46.592, 45.610, 10.809, 32.068, 24.095, 19.260, 11.501, *p* < 0.001). Overall, children and adolescents with muscle strength index >P80 had shorter reaction times, and those with muscle strength index <P20 had longer reaction times (Table 2).

**TABLE 2:** Comparison of the reaction times of children and adolescents with different muscle strength indexes in executive functions in rural areas of China.

Reaction time	MSI classification	*N*	RT (ms)		*F*-value	*P*-value
			***M***	**SD**		
**Inhibit function**						
Inconsistently	<P20	266	849.51	86.33	46.592	<0.001
	P20–80	802	803.96	76.25		
	>P80	267	788.01	76.77		
Congruent	<P20	266	827.75	86.65	45.610	<0.001
	P20–80	802	782.22	76.19		
	>P80	267	767.29	76.55		
Inhibitory function	<P20	266	21.77	5.62	2.075	0.126
	P20–80	802	21.74	5.76		
	>P80	267	20.95	5.64		
**Refresh memory function**						
1back	<P20	266	1061.17	324.53	10.809	<0.001
	P20–80	802	990.50	317.22		
	>P80	267	936.09	282.43		
2back	<P20	266	1283.02	320.90	32.068	<0.001
	P20–80	802	1165.57	302.77		
	>P80	267	1060.27	372.07		
**Cognitive flexibility**						
Conversion	<P20	266	1243.82	253.08	24.095	<0.001
	P20–80	802	1141.12	241.16		
	>P80	267	1106.44	246.15		
Size parity	<P20	266	790.75	95.38	19.260	<0.001
	P20–80	802	750.06	100.22		
	>P80	267	739.79	123.48		
Cognitive flexibility	<P20	266	453.07	220.46	11.501	<0.001
	P20–80	802	391.06	227.06		
	>P80	267	366.65	193.38		

MSI, muscle strength index; N, number; RT, reaction time; M, mean; SD, standard deviation; ms, millisecond.

After adjusting for relevant confounding variables (Model3), the results of multiple linear regression analysis showed that compared to those with muscle strength index <P20, those with muscle strength index >P80 inconsistently, congruent, 1back, 2back, and conversion, size parity, and cognitive flexibility reaction times were reduced by 51.243, 50.653, 100.527, 185.766, 105.641, 39.062, and 66.579 ms, respectively, with statistically significant differences (*p* < 0.001) (Table 3).

**TABLE 3:** Multiple linear regression analysis of muscle strength index and executive function in children and adolescents in rural areas of China (*n* = 1,335).

Reaction time	Estimates (95% confidence interval)		
	**Model 1**	**Model 2**	**Model 3**
**Inconsistently**			
<P20	0.000 (Reference)	0.000 (Reference)	0.000 (Reference)
P20–80	−45.558 (−56.448, −34.667)^b^	−45.607 (−55.673, −35.54)^b^	−38.854 (−49.000, −28.707)^b^
>P80	−61.502 (−74.836, −48.168)^b^	−59.643 (−71.986, −47.299)^b^	−51.243 (−63.749, −38.736)^b^
*p* for trend	<0.001	<0.001	<0.001
**Congruent**			
<P20	0.000 (Reference)	0.000 (Reference)	0.000 (Reference)
P20–80	−45.527 (−56.416, −34.637)^b^	−45.559 (−55.640, −35.478)^b^	−39.300 (−49.480, −29.121)^b^
>P80	−60.459 (−73.792, −47.126)^b^	−58.611 (−70.972, −46.250)^b^	−50.653 (−63.200, −38.107)^b^
*p* for trend	<0.001	<0.001	<0.001
**Inhibitory function**			
<P20	0.000 (Reference)	0.000 (Reference)	0.000 (Reference)
P20–80	−0.031 (−0.823, 0.761)	−0.049 (−0.839, 0.741)	0.385 (−0.404, 1.174)
>P80	−0.818 (−1.788, 0.152)	−0.797 (−1.766, 0.172)	−0.425 (−1.398, 0.547)
*p* for trend	<0.001	<0.001	<0.001
**1back**			
<P20	0.000 (Reference)	0.000 (Reference)	0.000 (Reference)
P20–80	−70.672 (−113.991, −27.354)^a^	−69.957 (−111.960, −27.955)^a^	−55.913 (−98.365, −13.461)
>P80	−125.088 (−178.125, −72.05)^b^	−121.175 (−172.677, −69.672)^b^	−100.527 (−152.852, −48.202)^b^
*p* for trend	<0.001	<0.001	<0.001
**2back**			
<P20	0.000 (Reference)	0.000 (Reference)	0.000 (Reference)
P20–80	−117.448 (−162.051, −72.846)^b^	−112.614 (−156.659, −68.569)^b^	−89.448 (−134.093, −44.803)^b^
>P80	−222.748 (−277.357, −168.138)^b^	−213.912 (−267.918, −159.905)^b^	−185.766 (−240.794, −130.739)^b^
*p* for trend	<0.001	<0.001	<0.001
**Conversion**			
<P20	0.000 (Reference)	0.000 (Reference)	0.000 (Reference)
P20–80	−102.702 (−136.650, −68.755)^b^	−106.130 (−138.770, −73.490)^b^	−81.026 (−113.748, −48.304)^b^
>P80	−137.382 (−178.945, −95.818)^b^	−135.374 (−175.396, −95.351)^b^	−105.641 (−145.972, −65.310)^b^
*p* for trend	<0.001	<0.001	<0.001
**Size parity**			
<P20	0.000 (Reference)	0.000 (Reference)	0.000 (Reference)
P20–80	−40.695 (−55.184, −26.207)^b^	−40.563 (−54.894, −26.232)^b^	−32.696 (−47.204, −18.188)^b^
>P80	−50.959 (−68.698, −33.220)^b^	−49.285 (−66.857, −31.712)^b^	−39.062 (−56.944, −21.181)^b^
*p* for trend	<0.001	<0.001	<0.001
**Cognitive flexibility**			
<P20	0.000 (Reference)	0.000 (Reference)	0.000 (Reference)
P20–80	−62.007 (−92.462, −31.552)^b^	−65.567 (−94.921, −36.214)^b^	−48.330 (−77.992, −18.668)^a^
>P80	−86.422 (−123.710, −49.134)^b^	−86.089 (−122.081, −50.097)^b^	−66.579 (−103.139, −30.018)^b^
*p* for trend	<0.001	<0.001	<0.001

Model 1 is an unadjusted variable; Model 2 adjusts for age, sex, only child, father’s education level, mother’s education level; Model 3 adjusts for breakfast, dairy products, duration of sleep, BMI, MVPA on the basis of Model 2 products, duration of sleep, BMI, MVPA.

^a^Indicates *p* < 0.05.

^b^Indicates *p* < 0.01.

After adjusting for relevant confounding variables (Model3), logistic regression analysis was performed using children and adolescents with muscle strength index >P80 as the control group. The results showed that the risk of 1back dysfunction was higher (OR = 1.764, 95% CI: 1.094, 2.843) in children and adolescents with muscle strength index <P20 (*p* < 0.05). The risk of 2back dysfunction was higher in children and adolescents with a muscle strength index <P20 (OR = 2.129, 95% CI: 1.329, 3.410) (*p* < 0.01). Compared with children and adolescents with muscle strength index >P80, the muscle strength index <P20 group had a higher risk of cognitive flexibility dysfunction (OR = 1.820, 95% CI:1.111, 2.982) (*p* < 0.05) (Table 4).

**TABLE 4:** Logistic regression analysis of muscle strength index and executive function in children and adolescents in rural areas of China (*n* = 1,335).

Executive dysfunction	Odds ratio (95% confidence interval)		
	**Model 1**	**Model 2**	**Model 3**
**Inhibitory function dysfunction**			
>P80	1.000 (Reference)	1.000 (Reference)	1.000 (Reference)
P20–80	1.339 (0.926, 1.936)	1.297 (0.893, 1.883)	1.321 (0.906, 1.926)
<P20	1.089 (0.691, 1.718)	1.058 (0.668, 1.678)	1.025 (0.638, 1.645)
*p* for trend	<0.001	<0.001	<0.001
**1back dysfunction**			
>P80	1.000 (Reference)	1.000 (Reference)	1.000 (Reference)
P20–80	1.449 (0.968, 2.169)	1.460 (0.969, 2.198)	1.332 (0.880, 2.015)
<P20	2.127 (1.346, 3.361) ^b^	2.119 (1.330, 3.376) ^b^	1.764 (1.094, 2.843) ^a^
*p* for trend	<0.001	<0.001	<0.001
**2back dysfunction**			
>P80	1.000 (Reference)	1.000 (Reference)	1.000 (Reference)
P20–80	0.999 (0.662, 1.505)	1.025 (0.678, 1.551)	1.012 (0.667, 1.536)
<P20	2.100 (1.335,3.304) ^b^	2.154 (1.363,3.404) ^b^	2.129 (1.329,3.410) ^b^
*p* for trend	<0.001	<0.001	<0.001
**Cognitive flexibility dysfunction**			
>P80	1.000 (Reference)	1.000 (Reference)	1.000 (Reference)
P20–80	1.271 (0.850, 1.900)	1.289 (0.849, 1.955)	1.131 (0.731, 1.749)
<P20	2.144 (1.364, 3.369) ^b^	2.419 (1.505, 3.887) ^b^	1.820 (1.111, 2.982) ^a^
*p* for trend	<0.001	<0.001	<0.001

Model 1 is an unadjusted variable; Model 2 adjusts for age, sex, only child, father’s education level, mother’s education level; Model 3 adjusts for breakfast, dairy products, duration of sleep, BMI, MVPA on the basis of Model 2 products, duration of sleep, BMI, MVPA.

^a^Indicates *p* < 0.05.

^b^Indicates *p* < 0.01.

## 4. Discussion

The purpose of this study was to investigate and analyze the association between muscle strength and executive function in children and adolescents in rural areas of China, in order to better promote the development of muscle strength and executive function. The association between muscle strength and executive function existed in children and adolescents in rural areas of China. Those with higher muscle strength had shorter executive function reaction times and were at lower risk of developing executive dysfunction. Our study included 1,335 children and adolescents aged 13–15 years from four different provinces in rural areas of China. The results of our study showed that the mean BMI (19.98 ± 2.48) kg/m^2^, whole-body muscle strength test results were: grip (29.98 ± 9.06) kg, pull up (2.91 ± 2.93) times, sit ups (29.80 ± 8.57), standing long jump (173.91 ± 31.48) cm.

Further analysis of the results of the muscle strength test revealed that the results of the grip test for rural children and adolescents in China were significantly lower than the average for children and adolescents aged 13–15 years in the United States (60.9 kg) (Ervin et al., 2014). The reason is that there is a correlation between BMI and muscle health, with leaner people tending to have less strength compared to normal weight children and adolescents (Rantanen et al., 2000). Compared to Spanish children and adolescents with similar average age and BMI, the muscle strength test values of children and adolescents in rural China were greater than those of Spanish adolescents with grip 24.71 kg, sit ups 24.90 times and standing long jump 150.99 cm. It may be related to the higher MVPA of children and adolescents in rural areas of China due to different economic levels, lifestyles and transportation modes (Martinez-Lopez et al., 2018). Comparing the results of muscle strength tests with those of Chinese domestic youth, we found that the grip test results were slightly higher than the average of 28.7 kg for 12–15 year olds, the sit ups were higher than the average of 22.53 times, and the standing long jump was lower than the average of 182.43 cm (Xu et al., 2020). This may be related to the data for 12-year-olds in the previous test results, as strength increases significantly with age in both children and adolescents (Martinez-Lopez et al., 2018). Comparison of the test results of our study with relevant national and international data revealed that, as in past studies, muscle mass strength can be used as a marker of nutritional status and physical function (Norman et al., 2011). Body weight and size were negatively correlated with physical performance tests, which were performed against gravity, such as standing long jump, sit ups, and pull up tests (Beunen et al., 1983; Malina et al., 1995). On the other hand, body weight and size are positively correlated with performance against external forces, such as the grip (Watson and O’Donovan, 1977).

One-way ANOVA and multiple linear regression analyses revealed that children and adolescents with high muscle strength had shorter executive function reaction times, i.e., higher levels of executive function. Our results are consistent with the findings of previous studies that muscle strength and executive function are correlated (Bruijn et al., 2018; Oberer et al., 2018; Mora-Gonzalez et al., 2019). Whereas muscle strength is associated with executive ability, the effects on the brain may be to alter the excitability of spinal motor neurons and to induce synaptogenesis in the spinal cord (Adkins et al., 2006). It is also likely that these adaptations in the brain may be mediated by physical activity (Hillman et al., 2008). After all, there is a correlation between increased levels of physical activity and improved muscle strength, and often those with higher physical activity also have higher muscle strength. These changes promote blood flow to the brain, which has a positive effect on the brain’s oxygen supply and nervous system, thus improving executive function (Shao et al., 2022).

Further logistic regression analysis also showed a strong association between poorer muscle strength and a higher prevalence of executive dysfunction. Previous studies have shown that high levels of muscle strength may indirectly improve health through beneficial effects on body composition and aerobic performance (Warburton et al., 2001). Muscle strength has little effect on maximal aerobic capacity, but they may improve submaximal exercise performance and thus reduce the risk of functional decline to indirectly reduce the risk of death (Hirvensalo et al., 2000). A review showed that resistance training particularly benefits cognition by increasing the expression of growth factors (i.e., BDNF, IGF-1) and changes in functional and structural properties of the brain (Herold et al., 2019). Notably, a meta-analysis did not observe a correlation between muscle strength and cognition, but reported that both muscle strength and muscle structure were associated with brain structure (Kilgour et al., 2014). Therefore, our future experimental studies need to verify whether muscle strength results from higher levels of executive function due to higher blood flow to the brain, higher oxygen-carrying capacity, and more excitable brain regions that control executive functions in the brain.

Several strengths exist in our study. On the one hand, our study is the first to investigate and analyze the association between muscle strength and executive function in children and adolescents in rural areas of China, which provides a reference for healthy development of children and adolescents in rural areas. In addition, our study uses a comprehensive muscle index reflecting muscle strength for analysis, which provides a more comprehensive response to the muscle strength level of children and adolescents. However, our study also has some limitations. First, the sample size of our study was relatively low, and the area investigated in the study was only in rural areas of four provinces in China, and the area studied was somewhat limited. Second, the control of covariates in our study was limited, such as the lack of investigation of cardiorespiratory endurance, sugary drinks, and smoking, and these deficiencies are to be remedied in future studies.

## 5. Conclusion

There is a close association between muscle strength and executive function in children and adolescents in rural areas of China. Children and adolescents with higher muscle strength in rural areas of China had shorter executive function reaction times and lower risk of executive function disorders. Based on our findings, effective measures should be taken in the future to improve muscle strength and executive function levels of children and adolescents in rural areas of China. Such as securing teaching time for physical education classes, securing time for extracurricular activities, strengthening health education, and conducting executive function training in order to promote the healthy physical and mental development of children and adolescents in rural areas of China. In addition, improving muscle strength and executive function in children and adolescents is a long-term process. It requires concerted efforts and joint efforts of families, schools and children and adolescents themselves to better achieve the improvement results.

## Data availability statement

The raw data supporting the conclusions of this article will be made available by the authors, without undue reservation.

## Ethics statement

The studies involving human participants were reviewed and approved the Human Ethics Committee of Huanghuai University (202105012). Written informed consent to participate in this study was provided by the participants or their legal guardian/next of kin.

## Author contributions

QZ, XH, and YW: conceptualization. QZ: data curation, funding acquisition, and visualization. QZ and XH: formal analysis, software, writing—original draft, and writing—review and editing. YW: investigation, methodology, validation, and resources. XH and YW: project administration and supervision. All authors have read and agreed to the published version of the manuscript.
